# An Assessment Framework for e-Mental Health Apps in Canada: Results of a Modified Delphi Process

**DOI:** 10.2196/10016

**Published:** 2018-07-09

**Authors:** Jennifer Zelmer, Krystle van Hoof, MaryAnn Notarianni, Trevor van Mierlo, Megan Schellenberg, Cara Tannenbaum

**Affiliations:** ^1^ Azimuth Health Group & University of Victoria Toronto, ON Canada; ^2^ Canadian Institutes of Health Research Institute of Gender and Health Montreal, QC Canada; ^3^ Mental Health Commission of Canada Ottawa, ON Canada; ^4^ Evolution Health Toronto, ON Canada; ^5^ Faculties of Medicine and Pharmacy Université de Montréal Montreal, QC Canada; ^6^ Institut Universitaire de Gériatrie de Montréal Centre de Recherche Université de Montréal Montréal, QC Canada

**Keywords:** mental health, mobile phone apps, consensus, Delphi process, evaluation framework, telemedicine

## Abstract

**Background:**

The number of e-mental health apps is increasing rapidly. Studies have shown that the use of some apps is beneficial, whereas others are ineffective or do not meet users’ privacy expectations. Individuals and organizations that curate, recommend, host, use, or pay for apps have an interest in categorizing apps according to the consensus criteria of usability and effectiveness. Others have previously published recommendations for assessing health-related apps; however, the extent to which these recommendations can be generalized across different population groups (eg, culture, gender, and language) remains unclear. This study describes an attempt by Canadian stakeholders to develop an e-mental health assessment framework that responds to the unique needs of people living in Canada in an evidence-based manner.

**Objective:**

The objective of our study was to achieve consensus from a broad group of Canadian stakeholders on guiding principles and criteria for a framework to assess e-mental health apps in Canada.

**Methods:**

We developed an initial set of guiding principles and criteria from a rapid review and environmental scan of pre-existing app assessment frameworks. The initial list was refined through a two-round modified Delphi process. Participants (N=25) included app developers and users, health care providers, mental health advocates, people with lived experience of a mental health problem or mental illness, policy makers, and researchers. Consensus on each guideline or criterion was defined a priori as at least 70% agreement. The first round of voting was conducted electronically. Prior to Round 2 voting, in-person presentations from experts and a persona empathy mapping process were used to explore the perspectives of diverse stakeholders.

**Results:**

Of all respondents, 68% (17/25) in Round 1 and 100% (13/13) in Round 2 agreed that a framework for evaluating health apps is needed to help Canadian consumers identify high-quality apps. Consensus was reached on 9 guiding principles: evidence based, gender responsive, culturally appropriate, user centered, risk based, internationally aligned, enabling innovation, transparent and fair, and based on ethical norms. In addition, 15 informative and evaluative criteria were defined to assess the effectiveness, functionality, clinical applicability, interoperability, usability, transparency regarding security and privacy, security or privacy standards, supported platforms, targeted users, developers’ transparency, funding transparency, price, user desirability, user inclusion, and meaningful inclusion of a diverse range of communities.

**Conclusions:**

Canadian mental health stakeholders reached the consensus on a framework of 9 guiding principles and 15 criteria important in assessing e-mental health apps. What differentiates the Canadian framework from other scales is explicit attention to user inclusion at all stages of the development, gender responsiveness, and cultural appropriateness. Furthermore, an empathy mapping process markedly influenced the development of the framework. This framework may be used to inform future mental health policies and programs.

## Introduction

The number and range of electronic health apps, including those targeting mental health, continues to expand [1,2]. Studies indicate that the use of some apps can be beneficial to mental health and many improve accessibility to mental health services [3]. According to a recent meta-analysis, mobile phone-based mental health apps can have positive effects on depressive symptoms, anxiety, and other mental health conditions [4]. Digital solutions, including mobile phone apps, can also help address some traditional barriers—cost, capacity, geography, and stigma—to mental health services [5]. However, research suggests that some apps are unsafe, ineffective, poorly documented, or do not meet users’ privacy and security expectations [6-8].

In Canada, supporting mental health and resilience through appropriate mobile health solutions is an area of growing policy interest, particularly given the rising rates of common mental health conditions [9] and unmet needs for services [10]. For example, *Changing Directions, Changing Lives*, the Mental Health Strategy for Canada, recommends increasing the use of e-mental health to reach more Canadians in need of support [11]. Accordingly, the Mental Health Commission of Canada has explored the use of these solutions in Canada and has also explored the associated opportunities and barriers to their use [5]. Likewise, the *Healthy and Productive Work Signature Initiative* of the Canadian Institutes of Health Research, which aims to support evidence-based interventions that foster healthy, meaningful, and productive work for all workers, recognizes e-mental health as a potential direction for improving the wellness of workers across the mental health continuum [12,13].

Currently, it can be time-consuming and difficult for potential users to assess the quality, safety, and evidence base of available health apps [3,14,15]. The private sector, government, academics, consumer groups, and others are trialing a range of strategies to tap into the benefits of eHealth while also addressing its attendant challenges. Regulatory, accreditation, market influence, educational, informational, and financial interventions are among the approaches that have been explored. For example, the United States Food and Drug Administration has established and updated regulatory guidance for mobile medical apps [16]. The Ontario Telemedicine Network has launched the public-facing Practical Apps website, which presents reviews of various health care apps; primary care providers can use this app to support their patients to better understand and manage their disease [17]. The National Health Service in the United Kingdom established a curated Apps Library, with apps assessed against a defined set of criteria, which was later rolled back following research that showed privacy and security gaps in a large proportion of the included apps [18]. However, this service has since been relaunched through a beta site [19]. Researchers in Canada and beyond are testing techniques to engage app users in the development process [20,21]. Likewise, Apple Inc. introduced specific requirements for medical apps that are made available through their App Store [22].

Several formal development strategies, rating scales, or assessment frameworks have been published to help raise standards on app quality, and efforts are underway to develop others [1,14,20,23-28]. At their core, most of these efforts depend on a structured assessment of apps against defined criteria. While there is some convergence on the technical criteria considered, there are also important differences between the approaches. The aims, scope, purpose, target audiences, and methods of assessment vary considerably. For instance, some initiatives consider factors such as the characteristics of the app developer or funder; their policies; and features of the app, its performance characteristics, and ongoing maintenance or updating requirements, while others do not. While these efforts have provided information and direction for this study, the authors were not able to find a single scale or framework that addressed the unique combination of cultural and political factors required for the Canadian context.

The well-documented variation in the app quality and safety requires a consistent and transparent assessment framework for apps applied at an organizational level or as a self-assessment for app developers. Results must be meaningful and trustworthy for potential app users with a wide variety of needs and perspectives. In Canada, improving and expanding ready access to—and use of—effective and appropriate e-mental health solutions, including mental health apps, holds promise as a key enabler for addressing mental health. The purpose of this study was to achieve consensus among a broad group of Canadian stakeholders on a comprehensive set of principles to guide the development of a framework for assessing e-mental health apps in Canada as well as future processes to implement such a framework. A secondary objective was to achieve consensus on a complementary set criteria to support and ground these guiding principles by providing informative and evaluative measures that could be applied as part of an assessment process. Methods and results are reported in alignment with the guidance for reporting results from Delphi processes developed by Boulkedid et al [29].

## Methods

### Study Design

**Figure 1:**
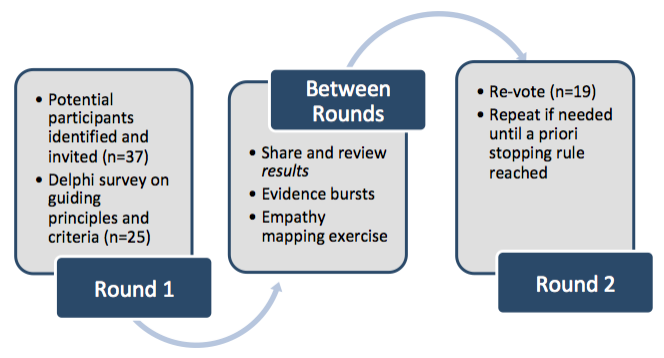
The modified Delphi process.

We used a modified Delphi process in a three-stage process with two voting rounds to reach consensus on guiding principles and criteria for a Canadian e-mental health app assessment framework (Figure 1).

Unlike the open-ended initial phase of a traditional Delphi process [30,31], a modified Delphi process provides a starting point for discussion. Experts are polled for their views on this starting point individually and anonymously through two or more rounds of voting. Results are provided to participants between rounds. The process concludes when predefined stopping points—usually a specified level of consensus—are reached. The mini-Delphi or estimate-talk-estimate approach adapts this technique for face-to-face meetings, allowing experts to interact between iterations of anonymous votes [32]. Figure 1 illustrates this approach as applied to this study. Ethics approval was not required for this study as it falls under an exemption for research conducted by faculty and staff as an outside professional activity (see exemption #8 on the University of Victoria website, the first author’s institution) [33].

### Participant Recruitment

We used a heterogeneous purposive recruitment strategy to seek diversity on the following three key variables: (1) the breadth of perspective with relevant expertise recognized by the Institute of Gender and Health at the Canadian Institutes of Health Research or the Mental Health Commission of Canada (app developers, app users, health care providers, mental health advocates, lived experience of mental health problems or illnesses either personally or as a family member, policy makers, researchers, and workplace or workforce expertise); (2) sex and gender; and (3) geographic distribution across Canada.

The project steering committee reviewed the distribution of potential participants against a structured template that showed distribution based on the abovementioned criteria. Relevant expertise was loosely defined as a characteristic of individuals recognized by their peers as having competence and experience in specific areas relevant to this project. For instance, researchers were selected on the basis of having received scientific funding or research productivity in the area of e-mental health; individuals with lived experience were part of a volunteer group that advises the Mental Health Commission of Canada.

Then, we purposefully selected and recruited potential participants via invitations from the Institute of Gender and Health at the Canadian Institutes of Health Research and the Mental Health Commission of Canada to maximize the breadth and depth of experience and expertise available from the participant group [34]. Invitations were distributed via emails, with follow-up by telephone or in-person as required. Those who indicated an interest in taking part then received an email with a link to the website hosting the first round of the Delphi study. This email included the rationale for seeking consensus on an assessment framework for e-mental health apps. Voluntary participation implied consent.

### Development of the Initial Set of Draft Principles and Criteria

First, we conducted a rapid review and environmental scan of pre-existing e-mental health app assessment frameworks found in the published and gray literature. Additional resources were identified and supplied by the modified Delphi participants. Alignment with Canadian culture and policies was considered. Gender responsiveness and cultural appropriateness were deemed to be two foundational and nonnegotiable elements, given Canada’s strong commitment to gender equality and cultural inclusiveness, particularly of indigenous people [35-37]. Delphi process organizers and the authors of this study felt it was an ethical imperative to include an app’s evidence base as a third foundational element of the framework in order to avoid the potential for harm from low-quality e-mental health apps.

Based on the findings, one of the authors (JZ) constructed a draft list of six other potential principles to guide the development and implementation of an e-mental health app assessment framework. Furthermore, 14 potential criteria were also identified, which aligned with the guiding principles and could be used as part of an app assessment process. The initial list was vetted by the steering committee (MN, MS, and KvH) for the project.

### Round 1 of the Modified Delphi Process

In Round 1 of the modified Delphi process, which took place between November 15 and 28, 2016, 25 stakeholders agreed to participate. The survey questions used to assess the initial list of principles and criteria were modeled after an app evaluation Delphi process led by researchers at the Imperial College in England [38] and pre-existing formats extracted from the rapid review and environmental scan of eHealth and e-mental health app assessment frameworks.

The survey was prefaced with three contextual questions:

1. Confirmation of the perspectives that the participant brought to the consensus process, as per the list above (participants could self-identify with multiple perspectives).
2. How they would rate currently available health apps in terms of overall quality, usability or user engagement, use of high-quality evidence from credible sources, and information security or privacy. Respondents were asked to endorse the applicability of each of these criteria using a 4-point scale (poor, fair, good, and excellent) plus a “don’t know” option.
3. Whether a framework for evaluating health apps is needed for consumers to identify apps of higher quality using a 5-point Likert scale (strongly disagree, disagree, neither agree nor disagree, agree, and strongly agree).

While gender and geographic diversity were considered in recruitment, we did not ask questions about these dimensions as part of the survey. This decision was taken to preserve the anonymity of responses, an important feature of a Delphi process. Inclusion of demographic variables in the survey would have made it possible to connect votes with the identity of at least some participants, which we sought to avoid. Likewise, we did not undertake a separate participant demographic survey to reduce the respondent burden.

The remaining survey questions assessed the initial list of 6 potential principles to guide the development of an e-mental health app assessment framework and the 14 potential criteria that might be included in such a framework. Criteria were classified as informative or evaluative. Informative criteria were defined as those where one answer is not “better” than another, but the information could be helpful to a user. Informative criteria would usually not be scored or have a minimum threshold for inclusion. Evaluative criteria were defined as those where directionality is clear (eg, whether more or less, presence or absence, is better). Evaluative criteria may have a minimum threshold for inclusion or be scored. We asked participants to rate the principles using a 5-point Likert scale (strongly disagree, disagree, neither agree nor disagree, agree, and strongly agree) and the criteria using a different 3-point scale (not necessary, desirable, and essential). Participants also had the opportunity to provide comments on and suggest additions to the guiding principles and criteria.

### Between Round Discussions, Evidence Presentations, and Empathy Mapping

A face-to-face meeting was held on November 28, 2016, at the Centre for Addiction and Mental Health (Toronto, Canada). Overall, 19 participants of the first modified Delphi process and all authors were in attendance. The event was facilitated by JZ, an experienced health services researcher with expertise in eHealth and a PhD in economics. She has previously led similar consensus-building exercises. Field notes were recorded by a research assistant.

At the beginning of the session, representatives from the Institute of Gender and Health of the Canadian Institutes of Health Research and the Mental Health Commission of Canada provided an overview of the policy context and rationale for this initiative. They specified that while a variety of approaches can be used for e-mental health app assessment, most depend on identifying a set of principles to guide the assessment process. The presenters reinforced that the scope of the exercise should be relevant to apps targeted at individuals and families seeking support to manage their own health, excluding apps targeted specifically at health professionals. Furthermore, clarification was provided that the framework could either be implemented by one or several organizations or as a self-assessment for app developers. Either way, the results of the assessment framework, once implemented, would be aimed at supporting the needs of members of the public as potential app users. App developers were considered a second important audience. Participants agreed that evidence base, gender responsiveness, and cultural appropriateness should be included as 3 foundational guiding principles of any Canadian assessment scale and need not be included in the consensus-building exercise.

Results from Round 1 of the modified Delphi process were presented using frequency distributions to summarize answers to survey questions with ordinal response scales. It was determined *a priori* that only principles and criteria from Round 1 voting with less than 80% agreement would be discussed during the meeting. Agreement was defined as endorsement of “strongly agree” or “agree” for the guiding principles and endorsement of “essential” or “desirable” for the criteria. Summaries of open-ended questions, including suggestions for changes or additions to principles or criteria, were also presented using quotes illustrative of the comments received. A facilitated iterative discussion led to modification, adaptation, removal, and addition to principles and criteria that did not initially secure 80% or greater agreement.

Furthermore, time was allocated for brief evidence presentations or “evidence bursts” from participant experts on opportunities for research related to eHealth apps, existing eHealth app assessment frameworks, and the digital health ecosystem. The purpose of the evidence bursts was to provide additional context and information to all participants on key considerations that might influence the selection of the guiding principles and criteria. Participants were allowed to ask questions after each presentation and discuss the relevance to the app assessment framework.

We used a facilitated persona empathy mapping exercise to ensure that a broad range of end users’ needs and perspectives were considered in the process [39,40]. Four personas were identified in advance, and participants added four more at the meeting (see Appendix 1). The eight personas represented a range of ages, sexes, genders, identities, cultures, geographic locations, and spectrum or severity of mental health conditions found across Canada. For each persona, meeting participants were encouraged to reflect and share the reasons why that individual might use an e-mental health app and what they imagined the use of such an app might achieve for each, according to individualized goals and needs. There was considerable discussion among meeting participants on these topics, including about how they might influence the principles and criteria. Consequently, several changes or additions were made to the text of the principles and criteria.

### Round 2 of the Modified Delphi Process

Following the presentations, evidence bursts, and empathy mapping exercise, the participants at the face-to-face meeting re-rated the principles and criteria (in some cases with changes or additions based on the discussion) using the same scales as used in Round 1. Voting was anonymous and took place using electronic tools and on paper. Participants did not have access to each other’s votes.

For Round 2 of the modified Delphi process, consensus was defined as 70% agreement, consistent with other studies of this type [41-43]. The intent was for items that did not achieve this consensus threshold to be included in a third postmeeting round of the Delphi process. This round, if required, would be conducted in the same manner as Round 1.

After Round 2 voting, participants shared key advice with policy makers on the directions ahead and provided feedback on the modified Delphi process. Following the session, a summary of the outcomes of the conversation was circulated to participants for review. We received no suggestions for amendments.

## Results

### Round 1 of the Modified Delphi Process

Participants in Round 1 of the modified Delphi self-identified as one or more of the following: app developers (5/24, 21%), app users (8/24, 33%), health care providers (8/24, 33%), mental health advocates (12/24, 50%), having lived experience of mental health problems as an individual living with such a problem or as a family member (8/24, 33%), policy makers (4/24, 17%), researchers (9/24, 38%), having workplace or workforce expertise (6/24, 25%), or having other relevant roles (6/24, 25%). There was one nonresponder. While participants could self-identify as having more than one role, many of them were explicitly recruited because of their lived experience of mental health problems.

In response to the contextual questions posed in Round 1, almost half of the participants (12/25, 48%) rated the overall quality of currently available health apps as poor or fair, whereas 24% (6/25) gave a good or excellent rating. The remainder of participants (7/25, 28%) indicated that they were uncertain of the overall quality of currently available health apps.

#### Guiding Principles

**Figure 2:**
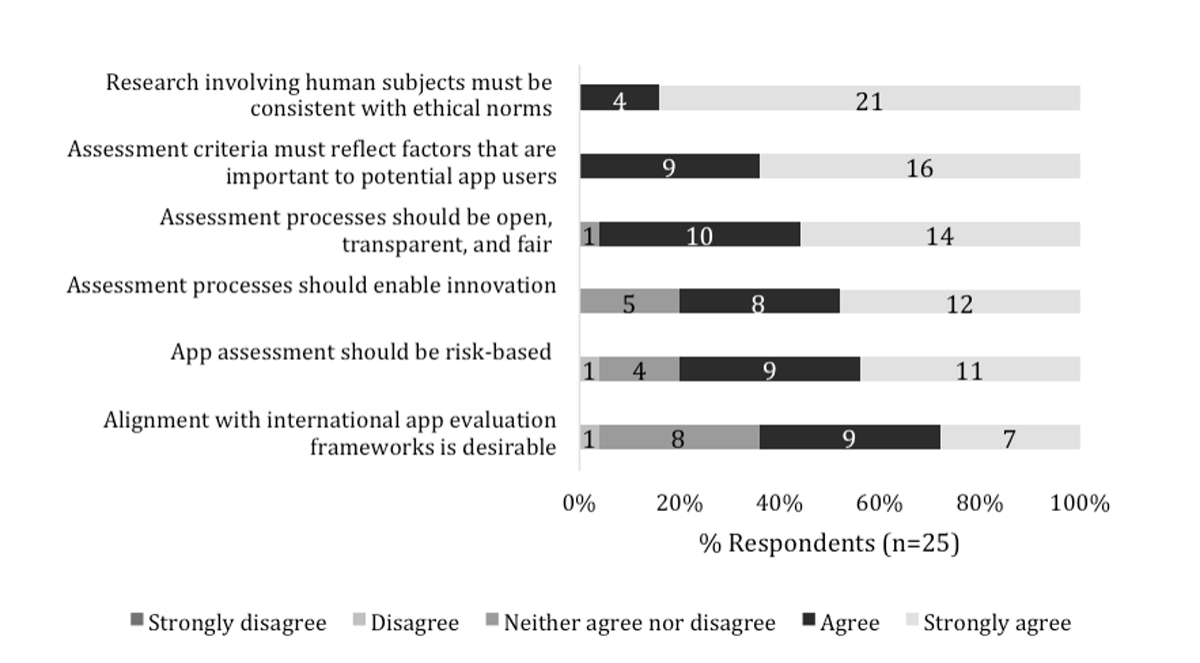
Rating of the initial guiding principles in Round 1.

Figure 2 illustrates the consensus ratings from the 6 guiding principles assessed during Round 1 voting. As 3 principles received “agree or strongly agree” ratings of at least 80% after Round 1, these were not discussed in detail prior to Round 2 voting; these included the principles that criteria should be user centered; processes should be open, transparent, and fair; and the research undertaken should reflect ethical norms. In addition, 2 principles representing the concepts that the apps enable innovation and be risk based received ratings of at least 70% but not 80%. Furthermore, the principle of international alignment received a rating of less than 70%.

#### Criteria

**Figure 3:**
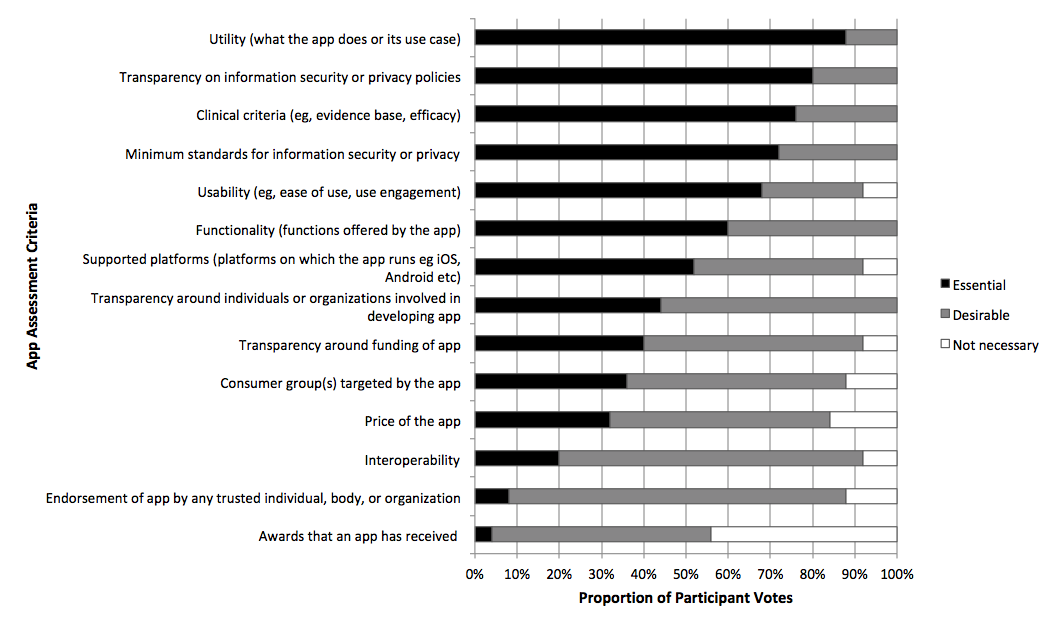
Round 1 rating of criteria for an e-mental health evaluation framework.

Round 1 voting included 14 potential criteria. Figure 3 lists the initial criteria assessed in Round 1 of the modified Delphi process and their endorsement by Delphi participants. All except one criterion met the essential or desirable inclusion consensus threshold. Only 2 of these—utility and transparency on information security or privacy policies—were endorsed as essential by at least 80% of participants. All participants voted on all criteria in this round, except one. There was one nonresponse for the “utility” criterion. The criterion of “awards that an app has received” was the only one that did not meet the consensus threshold for inclusion and was specifically discussed at the in-person meeting.

### Between Round Discussion, Evidence Presentations, and Empathy Mapping

#### Discussion: Guiding Principles

As per *a priori* decision rules, between rounds at the in-person meeting, participants discussed the 3 guiding principles that did not receive “agree or strongly agree” ratings of at least 80% in Round 1. In addition to the comments received as part of Round 1 open-ended questions, suggestions for revisions to the wording of these principles and the framework emerged throughout the discussion. One suggestion that was endorsed by all was to change the terminology from “evaluation framework” to “assessment framework” to reflect the breadth of the agreed-upon guiding principles and criteria. In addition, participants suggested that the 3 foundational criteria (evidence base, gender responsiveness, and cultural appropriateness) should be explicitly stated and added to the list of guiding principles.

#### Discussion: Criteria

Three additional criteria emerged through discussions among participants about the important perspectives and expertise that different stakeholders bring. These 3 additional criteria were added prior to Round 2 voting based on the feedback from participants. These potential criteria included user desirability, ideally presented in a way that can be stratified by the type of user; user inclusion in the development of the apps; and meaningful inclusion of a diverse range of communities in the testing of the apps. Participants underscored that user views must be sought, considered, and reflected throughout the app development process to ensure app quality and relevance. There was general agreement among participants that, at a minimum, stakeholder engagement in the development, implementation, and assessment of e-mental health apps should be expected.

Participants also discussed the appropriateness of the labels used for the list of criteria. There was general agreement that the “utility” criteria label should be replaced with the word “effectiveness” to clarify its meaning; this change was applied to the criteria list prior to Round 2 voting.

### Round 2 of the Modified Delphi Process

#### Guiding Principles

Following discussion, participants were asked to re-rate the relevant principles, with updated wording applied. All guiding principles reached at least 70% consensus in Round 2 (77%-100% agreed or strongly agreed). As a result, a third round of voting was not required.

A total of 9 guiding principles for an e-mental health app assessment framework were retained (Textbox 1). These included the 3 initial foundational principles: evidence base, gender responsiveness, and cultural appropriateness; 6 principles from the Round 1 list (3 with modifications based on participant input); and 1 new principle introduced on the basis of participant input at the in-person meeting.

#### Criteria

We asked participants to re-rate the relevant criteria, with updated wording applied. Table 1 shows the distribution of votes endorsing criteria as “essential,” “desirable,” or “not necessary” as well as the distribution of votes indicating how criteria should be approached—as either informative or evaluative. All criteria, except 2, reached at least 70% consensus, so a third round of voting was not required. The 2 criteria that were removed were “awards that an app has received” and “endorsement of the app by any trusted individual or body or organization” because participants expressed concern regarding the potential for bias in these criteria. The final criteria to be included in a Canadian e-mental health app assessment framework are listed in Textbox 2.

### Reaching Consensus on the Need for a Canadian e-Mental Health App Assessment Framework

In Round 1 and at the end of Round 2, we asked participants about the need for an app assessment framework to help consumers identify e-mental health apps of higher quality. Participants’ attitudes changed throughout the process. About two-thirds (17/25, 68%) of respondents in Round 1 agreed or strongly agreed with the need for the abovementioned framework. This number rose to 100% of participants after the in-person meeting, as assessed in Round 2 voting. For logistical reasons, some participants had to leave the in-person meeting prior to voting on this question, which resulted in n=13 for the Round 2 voting on this question.

**The final guiding principles for a Canadian e-mental health app assessment framework.**

**1. Evidence Based *(foundational principle)***

- Consideration must be given to the apps’ evidence base and effectiveness

**2. Gender Responsive *(foundational principle)***

- Apps must take into account sex and gender considerations

**3. Culturally Appropriate *(foundational principle)***

- Appealing to and inclusive of Canada’s diverse population

**4. User Centered**

- Assessment criteria must reflect the needs and expectations of potential app users

**5. Risk Based**

- App assessment should be risk based
- More detailed assessment is required for interventional apps, for example, drug dosing calculations or Web-based cognitive behavioral therapy, than for those focusing on general wellness support, for example, fitness trackers

**6. Internationally Aligned**

- App assessment framework should be informed by international experience or frameworks

**7. Allows for Development and Continual Improvement**

- The app assessment process should not impede the development or continual improvement of available apps

**8. Open, Transparent, and Fair**

- Assessment processes should be open, transparent, and fair

**9. Human Research Consistent with Ethical Norms**

- Research involving human subjects must be consistent with ethical norms
- Where an app is provided as part of a research study, Tri-Council Guidelines regarding Ethical Conduct for Research Involving Humans [37] must be followed.

**Table 1:** Round 2 criteria voting results.

Criteria	Distribution of endorsement			Approach to inclusion		
	Essential	Desirable	Not necessary		Informative	Evaluative
Effectiveness	18	0	0		4	19
Functionality	15	3	1		14	9
Clinical criteria	14	5	0		4	19
Interoperability	3	11	3		11	9
Usability	15	1	2		7	15
Information security transparency	18	1	0		4	19
Information security	16	2	0		4	17
Supported platforms	6	10	1		18	3
Audience	8	8	1		12	7
Developer transparency	9	6	0		18	5
Funding transparency	9	6	1		16	4
App price	6	10	2		19	2
User desirability	9	7	1		9	12
User inclusion	9	6	1		12	9
Meaningful inclusion	5	6	4		14	7
Endorsement of app^a^	3	8	7		6	4
Awards^b^	0	0	17		N/A^c^	N/A

^a^Did not meet the consensus threshold in Round 2.

^b^Did not meet the consensus threshold in Round 1; thus, omitted from informative versus evaluative criteria vote in Round 2.

^b^N/A: not applicable.

**The final criteria to be included in a Canadian e-mental health app assessment framework.**

**1. Effectiveness**

- What does the app do? What is its use case?

**2. Transparency of Information Security**

- Are the app’s security and privacy policies transparent and easy to find?

**3. Information Security**

- Does the app meet minimal standards for information security and privacy?

**4. Functionality**

- What are the functions offered by the app?

**5. Usability**

- Is the app easy to use? Do its intended users find it engaging?

**6. Clinical Criteria**

- What is the app’s evidence base? Is there evidence of its efficacy?

**7. Developer Transparency**

- Is there readily available information regarding the individuals and organizations involved in the development of the app?

**8. Funding Transparency**

- Is there readily available information regarding who funded the development of the app?

**9. User Inclusion**

- Were potential users involved in the development of the app?

**10. User Desirability**

- Have the intended users expressed a desire for the functionality provided by the app?

**11. Audience**

- Is it clear who the intended consumer group(s) is and what issues the app aims to address?

**12. Supported Platforms**

- What platforms does the app run on? (eg, iOS, Android, etc)

**13. App Price**

- How much does it cost to use the app? If it is not free, is it a one-time cost, subscription-based, or other?

**14. Meaningful Inclusion**

- Is information available on to what extent and how potential end users were involved in the development of the app?

**15. Interoperability**

- To what extent do users have the ability to move across different platforms (mobile and desktop) while maintaining profile preferences and information?

## Discussion

### Principal Findings

The Delphi process described in this study demonstrates that consensus was reached on a Canadian set of guiding principles and criteria for assessing the quality, effectiveness, and usability of evidence-informed e-mental health apps that adhere to ethical standards. The purpose of this study is to render transparent the process through which this framework was developed. Specifically, two rounds of a modified Delphi process, including the use of presentations, evidence bursts, and empathy mapping between the two rounds, led to an assessment framework incorporating 9 guiding principles and 15 supporting criteria.

These principles and criteria make up a framework that reflects Canadian priorities associated with the need for evidence-based solutions, transparency, gender responsiveness, cultural appropriateness, and user engagement at all levels of e-mental health app development and testing. Furthermore, iterative and interactive discussions solidified the perception that a framework is essential for meeting the promise and potential of using e-mental health apps as part of a broader strategy to improve mental health in Canada.

While the framework described here was developed with particular attention to the Canadian context and national priorities, the principles and results of our consensus process are generally consistent with other efforts and findings in this area. For example, a 2016 systematic review of methods used to assess mobile health apps found that researchers evaluated the app quality in 6 domains: scientific or clinical basis, functionality, usability, accountability, impact, and popularity; 80% (73/91) participants used measures drawn from one or more of these domains [14]. Table 2 illustrates how we see our framework as mapping onto these domains.

Given the potential global reach of apps and the growing evidence that many apps available today do not conform to the established guidelines or best practices [1], the need for an assessment framework is increasingly salient. Potential users and health care professionals who intend to recommend e-mental health apps will want a simple way of knowing the quality and other characteristics of apps available to them. Assessment frameworks are an important first step in addressing this need, but many challenges exist in achieving a widespread implementation and scale-up.

For instance, the Canadian framework described in this study was designed so that it could be applied at an organizational level or as a self-assessment for app developers. In the former case, a minimal standard of critical appraisal and the resources to undertake the analysis would be needed to research and assess the evidence base for an app, establish the characteristics of an app’s developers, and evaluate other criteria, such as cultural appropriateness. Indeed, even defining the process through which evidence base and clinical criteria will be assessed requires further investigation. In addition, an independent accreditation body could be tasked with this process to ensure quality and sustainability. While some Canadian organizations have assumed the aspects of this role (eg, via Canada Health Infoway’s certification program for privacy, security, and interoperability of digital health solutions), no organization has undertaken the full scope of assessment involved.

The Mental Health Commission of Canada is working on a fact sheet to disseminate the results of this consensus process broadly to those who might be able to use the information to affect change in a way that mental health apps are developed, recommended, and taken up. Given that health is provincially and territorially regulated in Canada, there is a potential for provincial and territorial health authorities to implement the framework, or an adapted version of it, within their jurisdictions as mental health apps become a more mainstream option to address barriers like affordability and lack of access in remote communities. The 2017 Canadian federal budget included Can $11 billion over 10 years aimed at supporting provinces and territories in improving mental health and home care services [44]. This investment, as well as the spotlight it has put on mental health services, creates an opportune moment to promote this framework as a tool to make e-mental health an effective part of evolving provincial and territorial strategies.

**Table 2:** How the Canadian framework maps onto 6 domains commonly used by researchers to evaluate the app quality.

Six domains	Canadian framework guiding principles	Canadian framework criteria
Scientific or clinical basis	Evidence based Human research consistent with ethical norms	Clinical criteria Meaningful inclusion
Functionality	Allows for development and continual improvement	Functionality Supported platforms Interoperability
Usability	Gender responsive Culturally appropriate User centered	Usability User inclusion
Accountability	Risk based Open, transparent, and fair	Transparency of information security Information security Developer transparency Funding transparency
Impact	Internationally aligned	Effectiveness App price
Popularity	N/A^a^	User desirability Audience

^a^N/A: not applicable.

Another question that must be addressed is how the assessment results would be presented. For instance, a key decision is whether evaluative criteria should be used to calculate an overall summary score for each app or there should be minimum acceptable thresholds for certain criteria. The *Mobile App Rating Scale* (MARS), developed in Australia and one of the most developed examples of an app evaluation tool, weighs all criteria equally [27]. MARS ratings average the mean scores from the following 5 subscales: engagement, functionality, esthetics, information, and subjective quality. Given that *evidence base* is only 1 of 7 items listed under the information subscale, an app could potentially receive a very high rating on the MARS scale without having a strong evidence base. This method does not appear to be consistent with the findings from our Canadian Delphi process, which highlighted the significance that stakeholders attach to factors such as evidence base, gender responsiveness, and cultural appropriateness. Thus, further research could be helpful in addressing these and related questions regarding how best to obtain assessment results that accurately and appropriately reflect the guiding principles of the framework.

Research could also help inform a number of other practical implementation challenges. For example, the landscape of global mental health apps is evolving rapidly. Participants in the Delphi process emphasized that an assessment framework and process should not impede the development or continual improvement of apps. In this context, one needs to consider factors such as the timeline and criteria for re-assessing apps as they evolve. Likewise, if apps that have been assessed can be accessed via an integrated listing or shared repository, who would curate the collection and how would need to be determined, as would how to encourage its use by the public and health professionals.

Many other countries around the world are grappling with similar issues related to the endorsement of e-mental health apps. Some countries have published recommendations for assessing health-related—including mental health-related—apps, but the extent to which they can be generalized across a variety of populations and their characteristics (eg, culture, gender, and language) remains unclear. This is one of the reasons why we used a process focused on the needs of stakeholders across Canada. While the diversity of our participants is a strength of this study, we solicited their feedback within a defined Canadian scope and perspective. Furthermore, there was a 20% attrition of participants who voted in the first (Web-based) and second (in-person) round of the Delphi process. This may have introduced selection bias for some of the principles and criteria retained in the final framework.

While the methods used in this study can be replicated readily, the outcomes that we obtained by consulting stakeholders from across Canada may or may not be transferable to other countries or contexts. As several of the factors considered in our process were aligned with a related process simultaneously underway in the United Kingdom, it may be possible to explore this question in the future when their results are available.

### Conclusions

Improving health gains and reducing risks from the use of e-mental health apps depends on tilting the balance toward solutions that are of higher quality and are more acceptable to potential users. To inform the efforts to achieve this goal, we used an innovative structured Delphi process that incorporated evidence bursts and empathy mapping to ask a diverse group of Canadian stakeholders about what factors were important in assessing e-mental health apps. The group reached consensus on 9 guiding principles and 15 criteria for an e-mental health app assessment framework that could be applied at an individual or organizational level to support them in meeting the needs and expectations of potential app users and other key stakeholders. This consensus has the potential to inform future research, policy, and programs at a still relatively early stage in the evolution of e-mental health solutions.

## Appendix

Multimedia Appendix 1 Personas for empathy mapping.

## Abbreviations

CIHR: Canadian Institutes of Health Research
MARS: Mobile App Rating Scale
