# Supplementary material for: An Assessment Framework for e-Mental Health Apps in Canada: Results of a Modified Delphi Process
Source: JMIR Mhealth Uhealth. 2018 Jul 9;6(7):e10016. doi: 10.2196/10016 (PMC6056739; doi:10.2196/10016)
Supplement: Multimedia Appendix 1 [file mhealth_v6i7e10016_app1.pdf]

Multimedia Appendix 1: Personas for Empathy Mapping

Initial Personas:

Four personas were designed specifically for this initiative and included in the pre-meeting package for participants. Stock photos were also used for each persona, but they cannot be reproduced here due to copyright restrictions. The personas were:

- **Ania** is a potential app user. She is moderately tech savvy, with experience in using apps for work and personal scheduling, listening to music, and playing games. Ania has a lived experience of moderate depression.

- **Jim** is an experienced app developer (for his own company and for others). He has heard about the e-mental health app ecosystem and is potentially interested in participating but also has other options for where to focus his efforts. Commercial viability and size of user community are important to Jim. He does not have a professional background in mental health but may partner with others who do.

- **Veena** is an experienced, qualified therapist with an active and diverse practice. She serves clients, mostly adults in the workforce, who live both locally and remotely. Veena is interested in how apps can help or harm her clients. She is personally relatively tech savvy.

- **John** is an experienced mental health researcher with an interest in understanding the determinants of mental health and mental illness, as well as outcomes of mental health interventions. He has heard about the e-mental health app ecosystem and is potentially interested in participating but also has other options for where to focus his efforts. John has not previously used data from apps for research or studied the use of apps.

Additional Personas

Based on input from participants at the face-to-face session, the following additional personas were added for empathy mapping:

- **Sheeba** has minimal experience with technology, lives alone in a rural/remote area, has multiple mental health conditions, is unemployed, and identifies as transgender.

- **Kathy** is responsible for mental health services for a provincial government. With limited budgets and multiple competing priorities, she is busy and feels stretched because of the multiple demands that she faces. Kathy is

indigenous.

- **Bob** is interested in wellness and health promotion. He has good days and bad days, but no formal mental health diagnosis. Bob is a white male and is the relative/friend of someone with lived experience of mental illness.

- **Mohammed** is the leader of a gay Muslim not-for-profit organization serving multilingual clients/community, many with a background of trauma and experience with multiple types of discrimination. He is personally not very tech savvy.
